# What Is Currently Known about Intramedullary Spinal Cord Abscess among Children? A Concise Review

**DOI:** 10.3390/jcm11154549

**Published:** 2022-08-04

**Authors:** Bartosz Szmyd, Redwan Jabbar, Weronika Lusa, Filip Franciszek Karuga, Agnieszka Pawełczyk, Maciej Błaszczyk, Jakub Jankowski, Julia Sołek, Grzegorz Wysiadecki, R. Shane Tubbs, Joe Iwanaga, Maciej Radek

**Affiliations:** 1 Department of Neurosurgery, Spine and Peripheral Nerves Surgery, Medical University of Lodz, 90-549 Lodz, Poland; 2 Department of Pediatrics, Oncology and Hematology, Medical University of Lodz, ul. Sporna 36/50, 91-738 Lodz, Poland; 3 Department of Sleep Medicine and Metabolic Disorders, Medical University of Lodz, 92-215 Lodz, Poland; 4 Department of Pathology, Chair of Oncology, Medical University of Lodz, 92-213 Lodz, Poland; 5 Department of Normal and Clinical Anatomy, Chair of Anatomy and Histology, Medical University of Lodz, Żeligowskiego 7/9, 90-752 Lodz, Poland; 6 Department of Neurosurgery, Tulane Center for Clinical Neurosciences, Tulane University School of Medicine, New Orleans, LA 70112, USA; 7 Department of Neurosurgery and Ochsner Neuroscience Institute, Ochsner Health System, New Orleans, LA 70433, USA; 8 Department of Neurology, Tulane Center for Clinical Neurosciences, Tulane University School of Medicine, New Orleans, LA 70112, USA; 9 Department of Anatomical Sciences, St. George’s University, Grenada FZ 818, West Indies; 10 Department of Surgery, Tulane University School of Medicine, New Orleans, LA 70112, USA

**Keywords:** intramedullary spinal cord abscess, ISCA, abscess, spinal cord tumor, antibiotics, dermal sinus, epidermoid cyst, dermoid cyst

## Abstract

Intramedullary spinal cord abscesses (ISCA) are rare. Typical symptoms include signs of infection and neurological deficits. Symptoms among (younger) children can be highly uncharacteristic. Therefore, prompt and proper diagnoses may be difficult. Typical therapeutic options include antibiotics and neurosurgical exploration and drainage. In this review, we analyze published cases of ISCA among children. Most pediatric cases were found to be under the age of 6 years. The typical symptoms included motor deficits in 89.06%, infection signs in 85.94%, and sensory deficits in 39.06%. Urinary dysfunction was observed in 43.75%, and bowel dysfunction in 17.19%. The predisposing factors included dermal sinuses, (epi)dermoid cysts, prior infection, iatrogenic disorder, and trauma. The most common pathogens were: *Staphylococcus aureus*, *Mycobacterium tuberculosis*, *Escherichia coli*, and *Proteus mirabilis*. The pediatric population has good outcomes as 45.93% of patients had complete neurological recovery and only 26.56% had residual neurological deficits. Fifteen (23.44%) had persistent neurological deficits. Only one (1.56%) patient died with an ISCA. In two (3.13%) cases, there were no details about follow-up examinations.

## 1. Introduction

**Figure 1:**
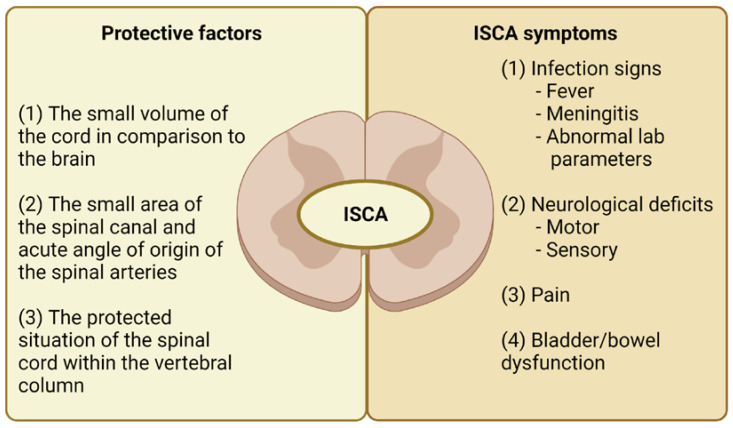
The protective factors and typical symptoms of intramedullary spinal cord abscess. Legend: ISCA—intramedullary spinal cord abscess.

The intramedullary spinal cord abscesses (ISCAs) remain a rare, albeit widely publicized entity since the first reported case in 1830 [1,2]. Their rarity may be explained by the following factors: (1) the small volume of the spinal cord compared to the brain, (2) the small area of the spinal canal and acute angle of origin of the spinal arteries, and (3) the protected condition of the cord within the vertebral canal (see Figure 1) [3].

The typical symptoms include infection signs (fever/meningitis), neurological deficits (motor and/or sensory), and also pain (see Figure 1). These symptoms among children, especially younger ones, can be highly uncharacteristic and regrettably, can be associated with significant mortality. Therefore, a rapid and proper diagnosis may be difficult. Typical therapeutic options include antibiotics and neurosurgical exploration and drainage [4].

In this review, we analyze the currently published cases of ISCA among children in the terms of basic demographic data, location, symptoms (ISCA signs, infection parameters, additional information), course, pathogens, comorbidities, treatment methods, and follow-up examinations.

## 2. Literature Search

**Figure 2:**
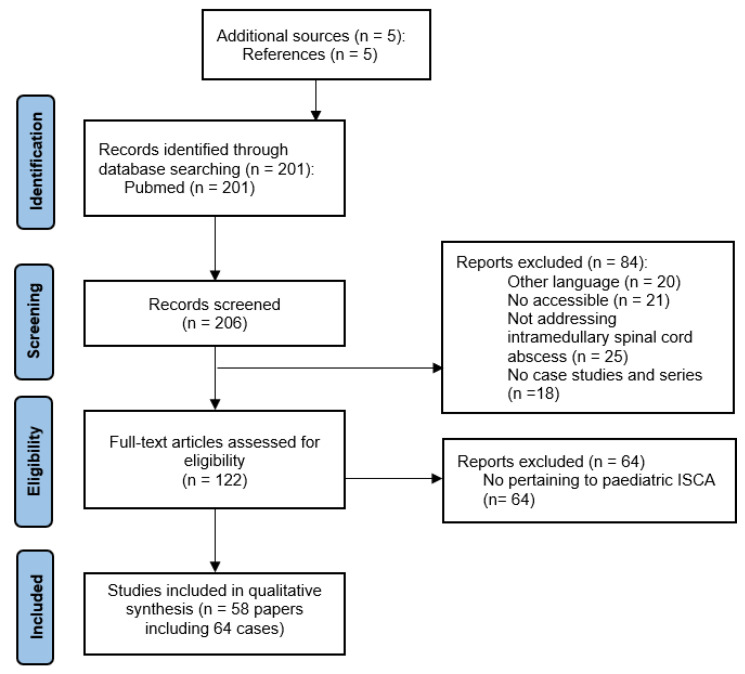
The flow-chart of publications included process.

Three of us (B.S., R.J., and W.L.) performed a screening of all the relevant original English language papers published in the Pubmed before 1 May 2022 using the following query: “((intramedullary) AND (spinal cord)) AND (abscess)”. As it is shown in Figure 2, we obtained 206 papers: 201 from the Pubmed database and 5 from additional sources). They were screened three times. In the case of any discrepancies between authors extracting data, the final decision was taken by the senior author (MR). In total, 122 papers potentially pertaining to the topic of the study were enrolled in the full-text assessment for eligibility. In these 122 papers, we identified 58 papers regarding pediatric ISCA with the description on 64 cases [5,6,7,8,9,10,11,12,13,14,15,16,17,18,19,20,21,22,23,24,25,26,27,28,29,30,31,32,33,34,35,36,37,38,39,40,41,42,43,44,45,46,47,48,49,50,51,52,53,54,55,56,57,58,59,60,61,62].

## 3. What Is Currently Known about Intramedullary Spinal Cord Abscesses in Children?

**Figure 3:**
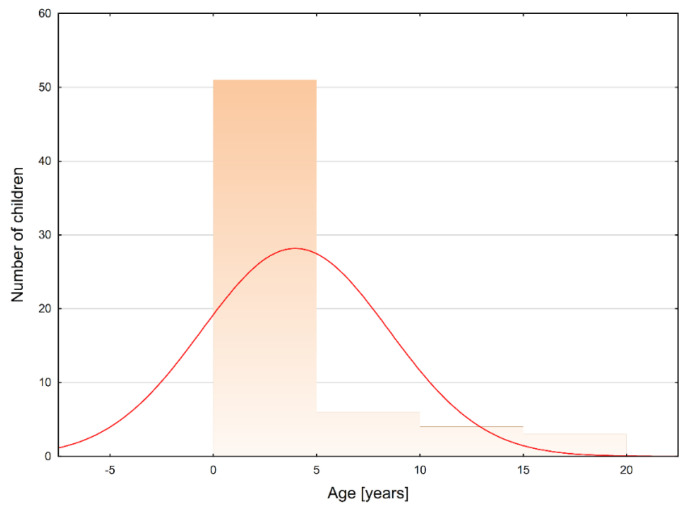
The age distribution among pediatric patients who developed intramedullary spinal cord abscesses (Shapiro−Wilk test: *p* < 0.001). Legend: red curve − expected normal distribution.

This diagnosis of ISCA was confirmed in 37 (57.81%) boys and 25 (39.06%) girls. In two cases (3.13%), there were no data regarding sex. The analyzed ages did not reveal a normal distribution (Shapiro–Wilk test; *p* < 0.001; see Figure 3). The median age of the patients was 2.00 years (IQR: 1.17–5.00). Boys were significantly older than girls: 3.60 (IQR: 1.42–6.00) vs. 1.33 (IQR: 1.00–2.25; *p* = 0.007).

### 3.1. ISCA Course and Localization

The course of ISCA can be divided into acute (<1 week), subacute (1–6 weeks), and chronic (>6 weeks) [44]. The most frequently observed manifestation was acute: 25 (39.06%) followed by 21 (32.81%) subacute cases. Chronic onset was observed in 13 (20.31%) cases. In five (7.82%) cases there were no detailed data. Neither sex (*p* = 0.350) nor age (R = −0.010, *p* = 0.940) affected the onset of ISCA [63].

**Figure 4:**
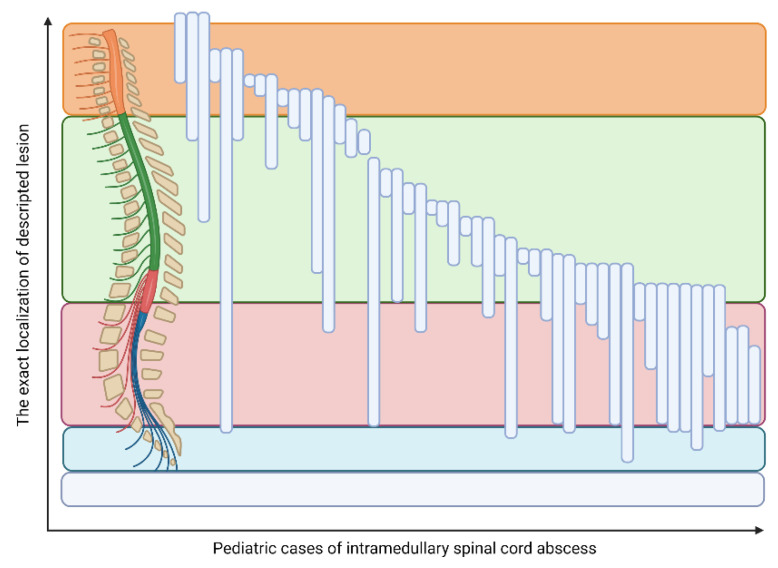
The localization of the intramedullary spinal cord abscess in children.

The exact location was identified in 60 cases (including seven holocords [28,29,35,51,52,54,56] and two isolated lesions in the conus medullaris [5,36]). The location of ISCA lesions in the remaining 51 cases is shown in Figure 4. The precise localization was not directly provided in four of the cases. In the newborn/infant group the spinal cord terminated most frequently at the level of L2/L3. As we age, the level of spinal cord termination is changing, and in the adolescent population, it was most often found at the level of the middle third of L1 and L1/L2 [64]. Therefore, it seems to be interesting that in 16 (25%) cases the abscess was observed below the L3 level. The possible reasons for these observations were found in 12 (75%) cases. There were distinguished the following causes: five (31.25%) cases of spina bifida [16,43,48,49,53], four (25.0%) cases of (possible) coexistence of ISCA and intradural extramedullary lesion [8,18,31,61], two (12.5%) cases of low conus medullaris [36,49]. Moreover, we identified one case of the following explanation: retained medullary cord [62], tethered cord [16], and mild thoracolumbar scoliosis with upper anal cleft [36]. Theoretically, the classification of the lesion within the terminal filum may be the next issue. Lesions in this localization are considered *intraspinal,* which may be in contradiction to the aforementioned end of the spinal cord.

### 3.2. Symptoms Present in ISCA Patients

Laboratory results indicative of inflammation/infection were identified in 55 (85.94%) patients. These included fever—39 (60.94%), abnormalities in laboratory tests (elevated white blood cell counts, C-reactive protein concentration, and erythrocyte sedimentation rate)—34 (53.13%), and symptoms of meningitis—12 (18.75%). Motor deficits were observed in 57 (89.06%) patients.

In the other cases, the following symptoms were noted, e.g., irritability [33], exaggerated lower and upper limb reflexes [43], and isolated fevers. Sensory deficits were noted in 25 (39.06%) patients. Moreover, urinary and bowel dysfunction were observed in 28 (43.75%) and 11 (17.19%) cases, respectively.

### 3.3. Predisposing Factors and Comorbidities

#### 3.3.1. Dermal Sinus Tracts

**Figure 5:**
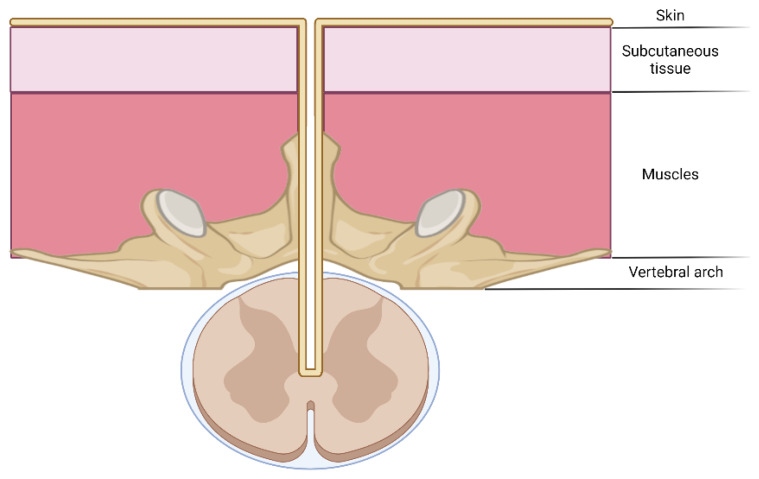
The schematic representation of a dermal tract as a predisposing factor for intramedullary spinal cord abscesses.

Congenital midline defects, as well as anatomic abnormalities of the spinal cord or vertebral column, are some of the key predisposing factors for ISCA. One of these is dermal sinus tracts (see Figure 5), an abnormality present at birth over the dorsal midline where an abnormal epithelialized connection from the skin tracks inwards toward the spine, especially in the lumbar (32–43%) and the lumbosacral regions (32–54%) [65]. Their prevalence is estimated at 1 in 2500 live births.

In our literature search, dermal sinus sinuses were observed in 35 (54.68%) children. The causative organisms among these patients include the microorganisms colonizing the skin surrounding the sinus tract openings [66].

#### 3.3.2. (Epi)dermoid Cyst

Epidermoid and dermoid cysts are two major variants of ectodermal-derived neural axis cysts [52]. Here we found three cases of this condition in ISCA patients [8,12,52]. Interestingly, these pathological entities can be related to a dermal sinus tract it is not mandatory [52].

#### 3.3.3. Spina Bifida

**Figure 6:**
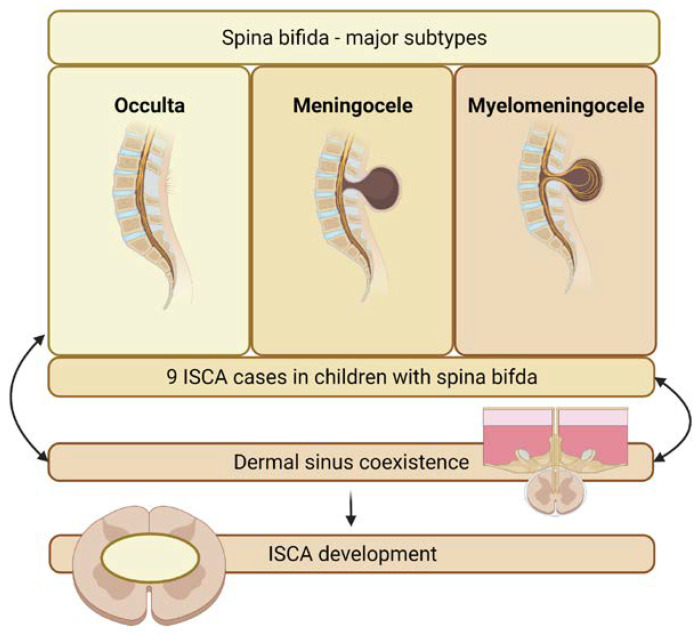
Relationship between spina bifida and intramedullary spinal cord abscess.

We identified nine cases of ISCA related to spina bifida (see Figure 6) [8,13,16,42,43,48,49,53]. In almost all of these cases, the presence of dermal sinus tracts was noted. Therefore, it should be assumed, that the true predisposing factor, dermal sinus tracts, is more frequently observed among patients with abnormalities of the ectodermal, mesenchymal, or neural crest derivatives such as myelomeningocele, lipomylomeningocele, and other forms of spina bifida occulta [42]. Perhaps a similar explanation can be given in the case of ISCA among adult patients born with talipes equinovarus [67,68].

#### 3.3.4. Prior Inflammation

Prior inflammation is a risk factor for developing ISCA. It may lead to a hematogenous or contagious spread of infection. The following scenarios were observed: general infection [30,34], respiratory system infection [14,24,27], maxillary sinus abscesses [33], Brucella infection [19], and long-term diarrhea [11]. Interestingly, there were noted some cases of previous tuberculosis, e.g., [22,33,41].

#### 3.3.5. Others

Other risk factors included iatrogenic ones as well as trauma. In our literature search, we have identified one case of ISCA which developed in the course of multiple attempts to perform a lumbar puncture and a second one due to spinal cord injury [44,47].

### 3.4. Available Treatments

Currently used treatments incorporate both neurosurgical management and anti-biotic/antifungal agents. Neurosurgeons may propose a (hemi)laminectomy with a myelotomy [4]. The abscess drainage with or without capsule removal may also be considered. In the cases of dermal sinuses, the dermal sinus tract should be identified and resected [36,59], and/or ligated [10]. Proper antimicrobial therapy depends on single cases beginning with empiric therapy and should be modified based on an antibiogram (see Section 3.5 for further information). In selected cases, glucocorticoids were administered to reduce edema [9,14,19,22,25,60].

### 3.5. Pathogens

Data regarding ISCA pathogens could not be obtained for four patients. In the other 60 cases, fifteen patients had culture-negative results (see Supplementary Table S1). Among those positive for just one microorganism, the most common pathogens were *Staphylococcus aureus* (8; 12.5%), *Mycobacterium tuberculosis* (6, 9.38%), *Escherichia coli* (4; 6.25%), and *Proteus mirabilis* (4; 6.25%). Moreover, *Brucella and Streptococcus* were identified in some cases. There were single cases of *Bacillus fusiformis*, *Enterobacter sakazakii*, *Finegoldia magna*, *Micrococcus sapproticus, Mycoplasma hominis, and Propionibacterium* (see Table S1). In 12 (18.75%) cases, there was more than one pathogen (see Table 1).

The antimicrobial therapy in *Staphylococcus aureus* ISCA cases was mainly six weeks of the administration of intravenous vancomycin in combination with other antibiotics (especially tazocin). The patients with *Escherichia coli* were treated with the usage of third-generation cephalosporin-based therapy. Finally, cases with *Proteus mirabilis* were treated as follows: one case using methicillin and chloramphenicol and a second case using ceftriaxone and clarithromycin, in the last two cases there were no data regarding antimicrobial drugs.

### 3.6. Follow-Up

The median time of follow-up examinations was six months (IQR: 2–18.25). The obtained follow-ups in the pediatric population seem to be optimistic: 29 (45.31%) patients revealed complete neurological recovery and 17 (26.56%) had residual neurological deficits. Fifteen (23.44%) had persistent neurological deficits. Just one (1.56%) child died in the course of ISCA [43]. In two cases (3.13%), there was detailed information about a follow-up examination (see Table 2).

## 4. Conclusions

ISCA remains a rare condition. Most pediatric cases are less than six years old. The typical symptoms include motor deficits in 89.06% of patients, infection signs in 85.94% of patients, and sensory deficits in 39.06% of patients. Moreover, urinary and bowel dysfunction was observed in 43.75%, and 17.19% of patients, respectively. Predisposing factors include dermal sinus tracts, (epi)dermoid cysts, prior inflammation, iatrogenic disorder as well as trauma. Currently used treatments incorporate both neurosurgical management and antimicrobial agents. In certain cases, glucocorticoids were administered. The most common pathogens were *Staphylococcus aureus*, *Mycobacterium tuberculosis*, *Escherichia coli*, and *Proteus mirabilis*. The most frequent antimicrobial treatment was six weeks of vancomycin in combination with other antibiotics (especially tazocin) for *Staphylococcus aureus*; third-generation cephalosporin-based therapy for *Escherichia coli*; and there was no consensus for *Proteus mirabilis*-related cases. At follow-up, 45.93% of patients revealed complete neurological recovery and 26.56% had residual neurological deficits. Fifteen (23.44%) had persistent neurological deficits. Just one (1.56%) child died of ISCA. In two (3.13%) cases, there were no details about follow-up examinations.

## Figures and Tables

**Table 1:** Microbiological examination among pediatric ISCA patients.

Microbiological Examination	Number
Culture-negative	15
*Staphylococcus aureus*	8
*Mycobacterium tuberculosis*	6
*Escherichia coli*	4
*Proteus mirabilis*	4
Others	11
No data	4
More than one pathogen	12

**Table 2:** Follow-up findings of pediatric ISCA patients.

Follow-Up	Number
Survived; complete neurological recovery	29
Survived, residual neurological deficits	17
Survived, persistent neurological deficits	15
Died	1
No data	2

## Data Availability

Not applicable.

## Supplementary Materials

The following supporting information can be downloaded at: https://www.mdpi.com/article/10.3390/jcm11154549/s1, Supplementary Table S1. The basic information about published cases of paediatric intramedullary spinal cord abscesses. Legend: N.D.—no data, Inflamm.—inflammation signs/parameters; Sex: F—female, M—male; Location of abscess: C—cervical, L—lumbar, S -sacral, T—thoracic; Symptoms: ND—neurological deficits, ND (M)—motor neurological deficites, ND (M + S)—motor and sensory neurological deficits, ND (S)—sensory neurological deficits.

## References

[1] Hood B., Wolfe S.Q., Trivedi R.A., Rajadhyaksha C., Green B. (2011). Intramedullary Abscess of the Cervical Spinal Cord in an Otherwise Healthy Man. World Neurosurg..

[2] Hart J. (1831). Case of Encysted Abscess in the Spinal Cord. Med.-Chir. Rev..

[3] Foley J. (1949). Intramedullary abscess of the spinal cord. Lancet.

[4] Chan C.T., Gold W.L. (1998). Intramedullary Abscess of the Spinal Cord in the Antibiotic Era: Clinical Features, Microbial Etiologies, Trends in Pathogenesis, and Outcomes. Clin. Infect. Dis..

[5] DiTullio M.V.J. (1977). Intramedullary spinal abscess: A case report with a review of 53 previously described cases. Surg. Neurol..

[6] Bean J.R., Walsh J.W., Blacker M.H. (1979). Cervical Dermal Sinus and Intramedullary Spinal Cord Abscess: Case report. Neurosurgery.

[7] Tewari M.K., Devi B.I., Thakur R.C., Pathak A., Khandelwal N., Kak V.K. (1992). Intramedullary spinal cord abscess: A case report. Child’s Nerv. Syst..

[8] Benzil D.L., Epstein M.H., Knuckey N.W. (1992). Intramedullary Epidermoid Associated with an Intramedullary Spinal Abscess Secondary to a Dermal Sinus. Neurosurgery.

[9] Manfredi M., Bozzao L., Frasconi F. (1970). Chronic intramedullary abscess of the spinal cord. J. Neurosurg..

[10] Gindi S.E., Fairburn B. (1969). Intramedullary Spinal Abscess as a Complication of a Congenital Dermal Sinus. J. Neurosurg..

[11] Bartels R.H., Gonera E.G., Van Der Spek J.A.N., Thijssen H.O.M., Mullaart R.A., Gabreels F.J.M. (1995). Intramedullary Spinal Cord Abscess—A Case Report. Spine.

[12] Çokça F., Meço O., Arasil E., Ünlü A. (1994). An intramedullary dermoid cyst abscess due to Brucella abortus biotype 3 at T11-L2 spinal levels. Infection.

[13] Rogg J.M., Benzil D.L., Haas R.L., Knuckey N.W. (1993). Intramedullary abscess, an unusual manifestation of a dermal sinus. AJNR Am. J. Neuroradiol..

[14] Hanci M., Sarioglu A.C., Uzan M., Işlak C., Kaynar M.Y., Oz B. (1996). Intramedullary Tuberculous Abscess: A case report. Spine.

[15] Dev R., Husain M., Gupta A., Gupta R.K. (1997). MR of multiple intraspinal abscesses associated with congenital dermal sinus. AJNR Am. J. Neuroradiol..

[16] Morandi X., Mercier P., Fournier H.-D., Brassier G. (1999). Dermal sinus and intramedullary spinal cord abscess. Child’s Nerv. Syst..

[17] Bavdekar S.B., Rao N., Kamat J.R. (1997). Intramedullary spinal cord abscess. Indian J. Pediatr..

[18] Chidambaram B., Balasubramaniam V. (2001). Intramedullary Abscess of the Spinal Cord. Pediatr. Neurosurg..

[19] Helvaci M., Kasirga E., Cetin N., Yaprak I. (2002). Intramedullary spinal cord abscess suspected of Brucella infection. Pediatr. Int..

[20] Tsurubuchi T., Matsumura A., Nakai K., Fujita K., Enomoto T., Iwasaki N., Nose T. (2002). Reversible holocord edema associated with intramedullary spinal abscess secondary to an infected dermoid cyst. Pediatr. Neurosurg..

[21] Simon J.K., Lazareff J.A., Diament M.J., Kennedy W.A. (2003). Intramedullary abscess of the spinal cord in children: A case report and review of the literature. Pediatr. Infect. Dis. J..

[22] Tanriverdi T., Kizilkilic O., Hanci M., Kaynar M.Y., Ünalan H., Oz B. (2003). Atypical intradural spinal tuberculosis: Report of three cases. Spinal Cord.

[23] Morimoto K., Takemoto O., Nakamura H., Takeuchi M. (2003). Spinal Dermal Sinus Associated with Intramedullary Abscess and Dermoid. Pediatr. Neurosurg..

[24] Dutton J.E.M., Alexander G.L. (1954). Intramedullary spinal abscess. J. Neurol. Neurosurg. Psychiatry.

[25] Guzel N., Eras M., Guzel D.K. (2003). A child with spinal intramedullary abscess. Child’s Nerv. Syst..

[26] Betty M., Lorber J. (1963). Intramedullary abscess of the spinal cord. J. Neurol. Neurosurg. Psychiatry.

[27] Yuceer N., Senoglu M., Arda M.N. (2004). Intramedullary spinal cord abscess in a 4-year old child. Acta Neurochir..

[28] Bunyaratavej K., Desudchit T., Pongpunlert W. (2006). Holocord intramedullary abscess due to dermal sinus in a 2-month-old child successfully treated with limited myelotomy and aspiration. J. Neurosurg. Pediatr..

[29] Kalia V., Vibhuti, Aggarwal T. (2007). Holocord intramedullary abscess. Indian J. Pediatr..

[30] Kamgarpour A., Izadfar M.-A., Razmkon A. (2008). Neglected intramedullary cord abscess in a 3-year old child: A case report. Child’s Nerv. Syst..

[31] Hung P.-C., Wang H.-S., Wu C.-T., Lui T.-N., Wong A.M.-C. (2007). Spinal Intramedullary Abscess with an Epidermoid Secondary to a Dermal Sinus. Pediatr. Neurol..

[32] Gerlach R., Zimmermann M., Hermann E., Kieslich M., Weidauer S., Seifert V. (2007). Large intramedullary abscess of the spinal cord associated with an epidermoid cyst without dermal sinus. Case report. J. Neurosurg. Spine.

[33] Du Plessis J., Andronikou S., Theron S., Wieselthaler N., Hayes M. (2008). Unusual forms of spinal tuberculosis. Child’s Nerv. Syst..

[34] Baradaran N., Ahmadi H., Nejat F., Khashab M., Mahdavi A., Rahbarimanesh A.A. (2008). Recurrent meningitis caused by cervico-medullary abscess, a rare presentation. Child’s Nerv. Syst..

[35] Tufan K., Cekinmez M., Sener L., Erdogan B. (2008). Infected Lumbar Dermoid Cyst Presenting with Tetraparesis Secondary to Holocord Central Lesion. J. Child Neurol..

[36] Al Barbarawi M., Khriesat W., Qudsieh S., Qudsieh H., Loai A.A. (2009). Management of intramedullary spinal cord abscess: Experience with four cases, pathophysiology and outcomes. Eur. Spine J..

[37] Houx L., Brochard S., Peudenier S., Hieu P.D., Rémy-Néris O. (2011). Recovery after Tetraplegia Caused by Dermal Sinus Infection: Intramedullary Abscess and Tetraparesis. Pediatr. Neurol..

[38] Mohindra S., Sodhi H.S., Aggarwal A. (2011). Management problems of intramedullary holocord abscess: An illustration in a pediatric case. Child’s Nerv. Syst..

[39] Malik N., Behari S., Ansari M.S., Jaiswal A.K., Gupta P., Jain M. (2011). An Intramedullary Tuberculous Abscess of the Conus in a 5-Year-Old Child Presenting with Urinary Dysfunction. World Neurosurg..

[40] Aggarwal M., Aggarwal K.C., Karamchand, Aggarwal A. (2011). Intramedullary spinal cord abscess masquerading as spinal tumor. Indian Pediatr..

[41] Khalid M., Khalid S., Mittal S., Ahmad U. (2012). Intramedullary tubercular abscess with syrinx formation. J. Pediatr. Neurosci..

[42] Chopra A., Patra B., Aneja S., Mukherjee S., Maheswari A., Seth A. (2012). Spinal Congenital Dermal Sinus Presenting as a Diagnostic Conundrum. Pediatr. Neurosurg..

[43] Bukhari E.E., Alotibi F.E. (2013). Fatal Streptococcus melleri Meningitis Complicating Missed Infected Intramedullary Dermoid Cyst Secondary to Dermal Sinus in a Saudi Child. J. Trop. Pediatr..

[44] da Silva P.S.L., Loduca R.D.D.S. (2013). Intramedullary spinal cord abscess as complication of lumbar puncture: A case-based update. Child’s Nerv. Syst..

[45] Nicola Z., Antonio C., De Tommasi A. (2014). Cervical dermal sinus complicated with intramedullary abscess in a child: Case report and review of literature. Eur. Spine J..

[46] Ramesh V.G., Karthikeyan K.V., Kitchanan S., Sriraman B. (2013). Holocord abscess in association with congenital dermal sinus. J. Pediatr. Neurosci..

[47] Whitson W.J., Ball P.A., Lollis S.S., Balkman J.D., Bauer D.F. (2014). Postoperative Mycoplasma hominis infections after neurosurgical intervention. J. Neurosurg. Pediatr..

[48] Kanaheswari Y., Lai C., Lope R.J.R., Azizi A.B., Zulfiqar M.A. (2015). Intramedullary spinal cord abscess: The result of a missed congenital dermal sinus. J. Paediatr. Child Health.

[49] Bhanage A., Katkar A., Ghate P., Ratta B. (2015). Intra-medullary tubercular abscess with spinal dysraphism: An unusual case. J. Pediatr. Neurosci..

[50] Papaevangelou G., Tsitsopoulos P.P., Flaris N., Iliadis C., Tsonidis C. (2015). Dermal Sinus Tract of the Thoracic Spine Presenting with Intramedullary Abscess and Cranial Nerve Deficits. Pediatr. Neurosurg..

[51] Kamat A.S., Thango N.S., Ben Husein M. (2016). Proteus mirabilis abscess involving the entire neural axis. J. Clin. Neurosci..

[52] Karaaslan B., Ülkü G., Ucar M., Demirdağ T.B., İnan A., Börcek A.Ö. (2016). Intramedullary dermoid cyst infection mimicking holocord tumor: Should radical resection be mandatory?—A case report. Child’s Nerv. Syst..

[53] Sahu R.N., Bhaisora K.S., Godbole C., Das K.K., Mehrotra A., Jayesh S., Behari S., Srivastava A.K., Jaiswal A.K. (2016). Delayed intramedullary abscess in operated case of spinal lipoma. J. Pediatr. Neurosci..

[54] Tassigny D., Fomekong E., Koerts G., Raftopoulos C. (2018). Intramedullary holocord abscess secondary to infected dermoid cyst. Acta Neurochir..

[55] Vankipuram S., Sahoo S.K., Srivastava C., Ojha B.K. (2017). Spinal cord abscess secondary to infected dorsal dermal sinus in an infant: Uncommon presentation of a known entity. BMJ Case Rep..

[56] Shaikh S., Joshi R. (2016). Pyomyelia presenting as acute flaccid paralysis. Oxf. Med. Case Rep..

[57] Verdier E.P., Konsol O., Portillo S. (2018). Intramedullary cervical abscess mimicking a spinal cord tumor in a 10-year-old girl: A case-based review. Child’s Nerv. Syst..

[58] Alsubaie S., Dolgum S., Binkhamis K., Alweijri I., Bugshan A., Alzamil F., Bakhshan A. (2019). Finegoldia magna causing intramedullary thoracic spinal cord abscess in an infant. Anaerobe.

[59] Bevan R., Leach P. (2021). Infected congenital cervical dermal sinuses leading to spinal cord abscess: Two case reports and a review of the literature. Child’s Nerv. Syst..

[60] Akimoto T., Hirose S., Mizoguchi T., Yokota Y., Hara M., Ishihara M., Morita A., Nakajima H. (2020). Ruptured long intramedullary spinal cord abscess successfully treated with antibiotic treatment. J. Clin. Neurosci..

[61] Sehgal R., Budnik E., Mallik A., Bonwit A., Leischner M. (2021). A Rare Case of an Intramedullary Spinal Cord Abscess Due to Escherichia coli in a Pediatric Patient. Child Neurol. Open.

[62] Matsubara Y., Murakami N., Kurogi A., Lee S., Mukae N., Shimogawa T., Shono T., Suzuki S.O., Yoshimoto K., Morioka T. (2022). Intramedullary abscess at thoracolumbar region transmitted from infected dermal sinus and dermoid through retained medullary cord. Surg. Neurol. Int..

[63] Chi Square Calculator—Up To 5x5, with Steps. https://www.socscistatistics.com/tests/chisquare2/default2.aspx.

[64] Van Schoor A.N., Bosman M.C., Bosenberg A.T. (2015). Descriptive study of the differences in the level of the conus medullaris in four different age groups. Clin. Anat..

[65] Foster M.T., Moxon C.A., Weir E., Sinha A. (2019). Dermal sinus tracts. BMJ.

[66] Chandran R.S., Bhanuprabhakar R., Sumukhan S. (2017). Intramedullary Spinal Cord Abscess: Illustration of Two Cases and Review of Literature. Indian J. Neurosurg..

[67] Maurice-Williams R.S., Pamphilon D., Coakham H.B. (1980). Intramedullary abscess--A rare complication of spinal dysraphism. J. Neurol. Neurosurg. Psychiatry.

[68] Hardwidge C., PalSingh J., Williams B. (1993). Pyomyelia: An intramedullary spinal abscess complicating lumbar lipoma with spina bifida. Br. J. Neurosurg..

