# Supplementary material for: What Is Currently Known about Intramedullary Spinal Cord Abscess among Children? A Concise Review
Source: J Clin Med. 2022 Aug 4;11(15):4549. doi: 10.3390/jcm11154549 (PMC9369492; doi:10.3390/jcm11154549)
Supplement: Supplementary file 1 [file jcm-11-04549-s001.zip › jcm-1793051-supplementary.pdf]

No	Age [years]	Sex	Onset	Location of abscess	Inflamm.	Symptoms	Comorbidities and/or predisposing factors	Mechanism of infection	Pathogens	Neurosurgical management	Pharmacological treatment	Outcome	PMID
1	1.00	M	acute	T12-S3	+	exaggerated lower and upper limbs reflexes	Spina bifida, dermal sinus and/or tract	Contiguous	S. melliri, B. fragilis	Myelotomy, dermal sinus/tract removal	ceftriaxone, vancomycin, metronidazole, meropenem	Died (cardiac arrest)	23292742
2	1.33	M	acute	Th1 downwards	+	ND (M)	Dermal sinus and/or tract	Contiguous	B-proteus, streptococcus	Myelotomy, dermal sinus/tract removal	erythromycin, ampicillin, and sulphonamides	Persistent ND	5800472
3	0.08	N.D.	acute	Conus	+	N.D.	Low conus, dermal sinus and/or tract	Contiguous	P. mirabilis	Myelotomy	antibiotics (not specified)	Persistent ND	329455
4	11.00	M	chronic	T8-L1	-	ND (M + S), pain, bladder dysfunction	N.D.	Cryptogenic	Culture negative	Myelotomy, abscess capsule excision	N.D.	Persistent ND	1394271
5	17.00	M	subacute	T11-L2	+	ND (M + S), pain, bladder dysfunction	dermoid cyst without sinus tract	Cryptogenic	B. abortus	Myelotomy, dermoid cyst/abscess excision	ceftriaxone, tetracycline, and rifampicin	Persistent ND	7843817
6	5.00	M	subacute	T10/11	-	ND (M), bladder & bowel dysfunction	N.D.	Cryptogenic	Culture negative	Myelotomy	antibiotics (not specified)	Persistent ND	10771868
7	1.17	M	subacute	T12-L3	+	ND(M+S), discharge from sinus	Dermal sinus and/or tract	Contiguous	P. acnes, P. oralis, P. magnus, V. parvula, A. meyeri, B. ureolyticus	Laminectomy, purulent content drainage, dermal sinus/tract removal	vancomycin, ceftriaxone, ampicillin, metronidazole	Persistent ND	12586985
8	1.17	N.D.	subacute	T8-10	+	ND(M+S)	N.D.	Cryptogenic	Culture negative	Myelotomy	cephalosporin, antituberculosis drugs (not specified?), and methylprednisolone	Persistent ND	13680294
9	2.67	M	chronic	T9-T11	+	ND (M+S)	systemic infection	Hematogenous	Culture negative	Myelotomy	vancomycin, ceftriaxone, amikacin, steroids (not specified?)	Persistent ND	17643248
10	0.67	F	acute	T11-L5	+	ND (M)	Low conus, dermal sinus and/or tract	Contiguous	St. aureus	Myelotomy, dermal sinus/tract removal	vancomycin, tazocin	Persistent ND	19172309
11	1.00	F	acute	L1-S1	+	ND (M)	Dermal sinus and/or tract	Contiguous	St. aureus	Myelotomy, dermal sinus/tract removal	vancomycin, tazocin	Persistent ND	19172309
12	1.50	M	subacute	T5-T6	+	ND (M), bladder dysfunction	Spina bifida, dermal sinus and/or tract	Contiguous	M. tuberculosis	Not performed	isoniazid, rifampicin, ethambutol, pyrazinamide, streptomycin	Persistent ND	23257833
13	1.00	F	subacute	T2-3	+	ND (M+S)	Multiple attempts to	Cryptogenic	Culture negative	Myelotomy	vancomycin, meropenem	Persistent ND	23559394

							lumbar puncture						
14	1.25	M	subacute	T9-S2	+	ND (M+S), bladder dysfunction	Spina bifida, dermal sinus and/or tract	Contiguous	E. sakazakii	Dermal sinus/tract removal	meropenem, metronidazole, teicoplanin, crystalline penicillin, cefotaxime, dexamethasone	Persistent ND	25099316
15	0.83	F	subacute	T6/7	+	ND (M), pus leakage	Dermal sinus and/or tract	Contiguous	Finegoldia magna	laminectomy, evacuation of the abscess, dermal sinus excision, samples for histopathology and microbiological tests	meropenem, ceftriaxone, and metronidazole	Persistent ND	30738869
16	3.42	M	N.D.	C4-Th4	+	ND (M)	N.D.	Cryptogenic	Gram-positive diplococci	Laminectomy	penicillin, sulphadimidine, chloramphenicol	Persistent ND	13967857
17	15.00	F	chronic	T7-10		ND (M + S)	N.D.	Cryptogenic	N.D.	Myelotomy	penicillin, streptomycin, and methylprednisolone	Residual ND	5457648
18	15.00	M	acute	C2-Th2	+	ND (M)	Dermal sinus and/or tract	Contiguous	P. mirabilis	Myelotomy, dermal sinus/tract removal	methicillin, chloramphenicol	Residual ND	471207
19	7.00	M	chronic	T7/8	+	ND (M+S), bladder dysfunction	Interstitial infiltration of the left lung	Hematogenous	Culture negative	Myelotomy	isoniazid, rifampin, streptomycin, and methylprednisolone	Residual ND	8882702
20	3.00	M	acute	T12-S1	+	ND (M+S)	N.D.	Cryptogenic	Culture negative	Myelotomy, abscess capsule excision	N.D.	Residual ND	11275786
21	1.00	F	chronic	T12-S1	+	ND (M+S), bladder & bowel dysfunction	Dermal sinus and/or tract, erythema, meconium aspiration syndrome	Contiguous	Culture negative	Myelotomy	Cefotaxime, panipenem, and y-globulin	Residual ND	12422041
22	0.08	F	acute	Lx	-	ND (M), pus leakage	Dermal sinus and/or tract	Contiguous	N.D.	Puncture, myelotomy	antibiotics (not specified)	Residual ND	12944707
23	1.50	F	acute	C6-Conus	+	ND(M), bladder & bowel dysfunction	Small cutaneous nevus	Contiguous	P. mirabilis, E. coli	Myelotomy	clindamycin, cefotaxime, meropenem, trimethoprim	Residual ND	17877274
24	2.00	F	acute	C5-T1	+	ND (M)	Cervical dimple and dermal sinus and/or tract	Contiguous	St. aureus	Myelotomy, dermal sinus/tract removal	vancomycin, tazocin	Residual ND	19172309

25	6.00	M	chronic	C5-T11	N.D.	ND (M+S), bladder dysfunction	scoliotic deformity	Cryptogenic	St. aureus	Myelotomy	ceftriaxone, amikacin	Residual ND	22138908
26	1.50	F	chronic	T4-L5	+	ND(M+S), bladder dysfunction	Dermal sinus and/or tract	Contiguous	E. coli, P. aeruginosa	Myelotomy	antibiotics (not specified)	Residual ND	24470811
27	1.25	F	N.D.	Brainstem-T8	+	ND (M)	Dermal sinus and/or tract, septic state	Contiguous	Corynebacterium and peptococcus species	Myelotomy, dermal sinus/tract removal	Cephalosporin, ciprofloxacin, vancomycin, colistin, and meropenem	Residual ND	26458220
28	1.25	F	chronic	L1-S2 -> holocord (after first surgery)	N.D.	ND (M+S), bladder dysfunction	Dermal sinus and/or tract	Contiguous	E. coli	Myelotomy, dermoid cyst excision and dermal sinus/tract removal	N.D.	Residual ND	29185056
29	1.00	M	acute	C7-Th2	+	ND(M), visible abscess (over scapula)	Dermal sinus and/or tract	Contiguous	Culture negative	Myelotomy, abscess capsule excision	antibiotics (not specified)	Residual ND	29269365
30	4.00	M	chronic	Th10-S1	+	ND (M+S), bladder & bowel dysfunction	Dermal sinus and/or tract, lumbosacral meningocele (state after prophylactic surgery)	Contiguous	E. coli	Myelotomy	cefotaxime, vancomycin	Residual ND	10361972
31	2.25	F	acute	T10-L1	+	ND (M), pain, bladder & bowel dysfunction	Uncertain	Cryptogenic	Culture negative	Myelotomy	sulphadimidine, penicillin, and cortisone	Residual ND	13967857
32	3.00	F	subacute	Cervicomedullary-Upper lumbar	+	ND (M)	Dermal sinus and/or tract	Contiguous	St. aureus	Dermal sinus/tract removal, pus aspiration	antibiotics (not specified)	Residual ND	9127042
33	3.00	M	subacute	T10-L5	+	ND (M)	Spinal lipoma, lipomeningocele	Cryptogenic	Culture negative	Myelotomy	antibiotics (not specified)	Residual ND	27857795
34	4.50	M	subacute	T1-3	+	ND (M + S), bladder dysfunction	Previous infection (pulmonary infection)	Hematogenous	B. fusiformis	Myelotomy	Streptomycin, penicillin	Complete neurological recovery	13212422
35	2.00	F	acute	L2-L5 [L2-L3 with extramedullary part: L3-L5]	-	ND(M), pain, bladder & bowel dysfunction	Dermal sinus and/or tract, epidermoid cyst, spina bifida occulta	Contiguous	E. coli, P. mirabilis, B. fragilis	Myelotomy, epidermoid tumor excision and dermal sinus/tract removal	ampicillin, metronidazole	Complete neurological recovery	1738439

36	2.00	F	acute	L1-L3	+	ND (M)	Spina bifida, dermal sinus and/or tract	Contiguous	E. coli, Bacteroides fragilis, P. mirabilis	Myelotomy	antibiotics (not specified)	Complete neurological recovery	8279336
37	5.00	M	acute	Th8-Th9	+	ND (M + S), bladder dysfunction	Diarrhoea for 3 weeks	Cryptogenic	Culture negative	Myelotomy	amoxicillin, ceftazidime	Complete neurological recovery	7638666
38	15.00	F	acute	T11/12	+	ND (M+S), pain	Brucella infection	Hematogenous	Brucella	Myelotomy	streptomycin, doxycycline, rifampicin, ceftriaxone, and methylprednisolone	Complete neurological recovery	12139575
39	7.00	M	acute	Th7	+	ND (M+S), pain, bladder dysfunction	Tuberculosis	Hematogenous	M. tuberculosis	Hemilaminectomy, pus aspiration	isoniazid, rifampicin, streptomycin, and methylprednisolone	Complete neurological recovery	12815372
40	4.00	M	subacute	T10	+	ND (M+S)	Previous infection (upper respiratory tract)	Cryptogenic	M. sapproticus	Myelotomy	teicoplanin, sulbactam, ampicillin, ornidazole, and dexamethasone (preoperative)	Complete neurological recovery	15338340
41	0.17	M	subacute	Holocord	-	ND (M), discharge from sinus, bowel dysfunction	Dermal sinus and/or tract	Contiguous	St. aureus	pus aspiration, dermal sinus and/or tract removal	cloxacillin	Complete neurological recovery	16619639
42	1.50	M	subacute	Holocord	+	ND (M), bladder dysfunction	Dermal sinus and/or tract	Contiguous	N.D.	Myelotomy	antibiotics (not specified)	Complete neurological recovery	17595507
43	1.42	M	acute	T12-L5	+	ND(M+S), bladder & bowel dysfunction	Dermal sinus and/or tract	Contiguous	Staphylococcus, Peptostreptococcus, Prevotella	Myelotomy	vancomycin, ceftriaxone, metronidazole	Complete neurological recovery	17675033
44	7.00	M	subacute	Medulla-C4	+	meningitis, no ND	Previous infection (systemic)	Cryptogenic	St. aureus	N/A	ceftriaxone, vancomycin, and dexamethasone	Complete neurological recovery	18350307
45	6.00	M	acute	Medulla-L2	+	ND (M+S), bladder dysfunction	Previous meningitis, dermal sinus and/or tract	Contagious	Culture negative	Conus puncture, removal of derma sinus and/or tract	Cefotaxime, metronidazole, vancomycin	Complete neurological recovery	18660476
46	5.00	M	acute	L1-L5	+	ND(M), bladder & bowel dysfunction	Mild thoraco-lumber scoliosis and upper anal cleft	Unclear	St. aureus	Laminectomy	vancomycin	Complete neurological recovery	19172309

47	1.33	M	subacute	C2-S1	+	ND (M), bladder dysfunction	Dermal sinus and/or tract	Contiguous	P. oralis, P. asaccharolyticus	Laminectomy, abscess drainage, dermal sinus resection	ceftriaxone, metronidazole, vancomycin, cefotaxime, steroid bolus	Complete neurological recovery	21310342
48	5.00	M	subacute	T11-L1	+	bladder dysfunction, pain	bladder dysfunction	Hematogenous	M. tuberculosis	Myelotomy	rifampicin, isoniazid, ethambutol, and pyrazinamide	Complete neurological recovery	22251511
49	5.00	F	acute	T12- L1	+	ND (M+S)	N.D.	Cryptogenic	N/A	Drainage	ceftriaxone, cloxacillin, amikacin, prednisolone	Complete neurological recovery	22253156
50	0.75	M	N.D.	C4-C6	+	ND (M)	Dermal sinus and/or tract	Contiguous	P. mirabilis	Myelotomy, dermal sinus/tract removal	Ceftriaxone, Claritromycin	Complete neurological recovery	23907309
51	17.00	M	subacute	Medulla-T3	+	ND(M)	Trauma, spinal injury, dermal abrasion	Contiguous	M. hominis	Myelotomy	vancomycin, moxifloxacin, doxycycline, moxifloxacin, piperacillin, tazobactam, and meropenem	Complete neurological recovery	24856879
52	0.33	F	chronic	T11-S4	+	ND(M), bladder dysfunction	Spina bifida, low conus, dermal sinus and/or tract	Cryptogenic	M. tuberculosis	Myelotomy, dermal sinus/tract removal	anti-tubercular treatment (not specified)	Complete neurological recovery	25878753
53	2.00	M	acute	Holocord	+	ND (M)	Septic shock, dermal sinus and/or tract	Hematogenous	P. mirabilis	Laminectomy, drainage, dermal sinus/tract removal	N.D.	Complete neurological recovery	26960264
54	0.83	F	subacute	Holocord	+	ND (M), bladder dysfunction	Dermal sinus and/or tract	Contiguous	Culture negative	Pyomyelia drainage, dermal sinus and/or tract removal	vancomycin, meropenem, ceftriaxone	Complete neurological recovery	29497549
55	1.00	M	subacute	Holocord	+	ND (M), bladder dysfunction	dermoid cyst, recurrent urinary tract infections	Contiguous	E. coli	Partial laminectomy, abscess irrigation	Ceftriaxone, metronidazole, amikacin, carbapenem	Complete neurological recovery	27184560
56	10.00	F	acute	C2-C6	+	ND (M)	N.D.	Cryptogenic	S. viridans	Myelotomy	antibiotics (not specified)	Complete neurological recovery	29938303
57	17.00	M	acute	T5-12	+	ND (M+S)	Maxillary sinus abscess, bronchiectasis, chronic bronchitis	Hematogenous	S. intermedius	N.D.	ceftriaxone, vancomycin, ampicillin, meropenem, and methylprednisolone	Complete neurological recovery	33246901

58	2.00	M	chronic	L2-L4	+	ND(M)	Dermal sinus and/or tract	Contiguous	E. coli	Firstly: laminectomy and open biopsy. Then, second laminectomy, drainage	ceftriaxone	Complete neurological recovery	34124280
59	0.05	F	subacute	L3-5	+	bladder & bowel dysfunction	Retained medullary cord, dermal sinus and/or tract	Contiguous	S. anginosus, P. bivia, E. faecalis	laminotomy L3-L5, durotomy, resection of C-LS as a column, rostral incision, and pus irrigating	ampicillin, cefotaxime, vancomycin, meropenem, linezolid, and immunoglobulin (i.v.)	Complete neurological recovery	35242420
60	3.00	F	N.D.	C5-6	-	ND (M)	Dermal sinus and/or tract	Contiguous	Culture negative	dermal sinus dissection, sampling for culturing and for biopsy	antibiotics (not specified)	Complete neurological recovery	32632578
61	1.33	F	N.D.	C4	+	nonspecific	Dermal sinus and/or tract	Contiguous	Propionibacterium species	dermal sinus excision, sampling for culturing and for biopsy	antibiotics (not specified)	Complete neurological recovery	32632578
62	1.25	F	chronic	T10-Sx	+	ND (M+S), bladder & bowel dysfunction	Spina bifida, tethered cord, dermal sinus and/or tract (state after prophylactic surgery)	Contiguous	E. faecalis	Myelotomy	cefotaxime, amoxicillin, and vancomycin	Complete neurological recovery	10361972
63	3.60	M	acute	C5-T2	+	irritability, no other neurological findings	Tuberculosis	Hematogenous	M. tuberculosis	Antibiotics (not specified)	N.D.	N.D.	17987300
64	4.00	M	chronic	T6 - Conus	+	ND (M)	Tuberculosis	Hematogenous	M. tuberculosis	Myelotomy	antibiotics (not specified)	N.D.	22837784

Supplementary Table S1. The basic information about published cases of paediatric intramedullary spinal cord abscesses. Legend: N.D. - no data, Inflamm. - inflammation signs/parameters; Sex: F - female, M - male; Location of abscess: C - cervical, L - lumbar, S -sacral, T - thoracic; Symptoms: ND - neurological deficits, ND (M) - motor neurological deficites, ND (M + S) - motor and sensory neurological deficits, ND (S) - sensory neurological deficits.
